# Supplementary figures and images for: Stochastic models allow improved inference of microbiome interactions from time series data
Source: PLoS Biol. 2024 Nov 21;22(11):e3002913. doi: 10.1371/journal.pbio.3002913 (PMC11620570; doi:10.1371/journal.pbio.3002913)

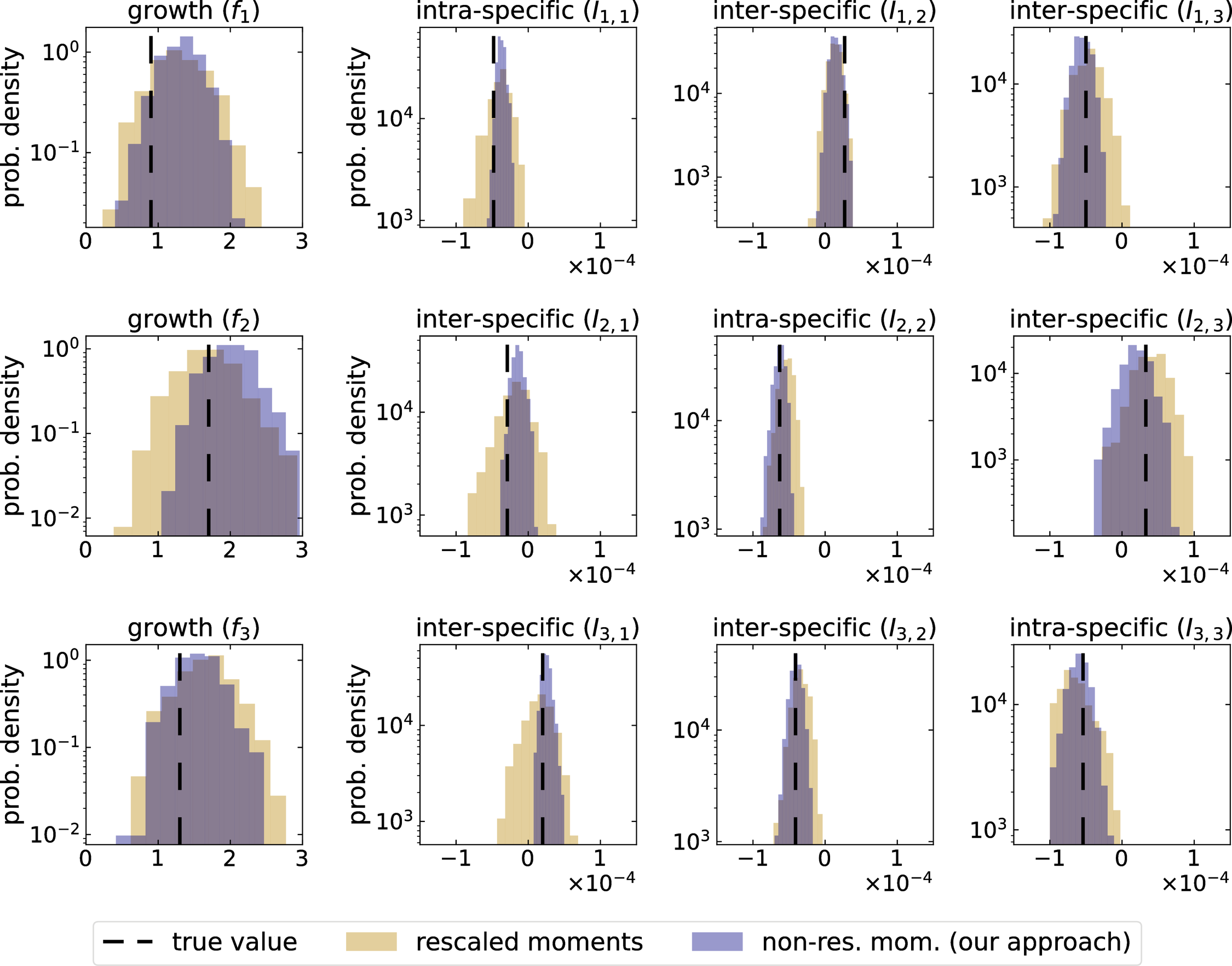

Supplement: S1 Fig — We inferred all parameters from relative abundance simulated data as shown in Fig 2. However, for the distance metric between model and data, Eq (4), statistical moments were rescaled or not. While for absolute abundance, the second-order moments and co-moments are naturally larger than the first-order moments, for relative abundance data, the opposite is true. Rescaling the moments can modify their importance during the inference process. To test this, we took the square root (of the squared errors) of second-order moments and co-moments for absolute abundance, and of first-order moments for relative abundance data. The posteriors of rescaled and non-rescaled moments largely overlap, with non-rescaled moments (our approach in the other figures) leading to more certainty. The data underlying this figure can be found in https://doi.org/10.5281/zenodo.13958305. (TIF) [file pbio.3002913.s001.tif]

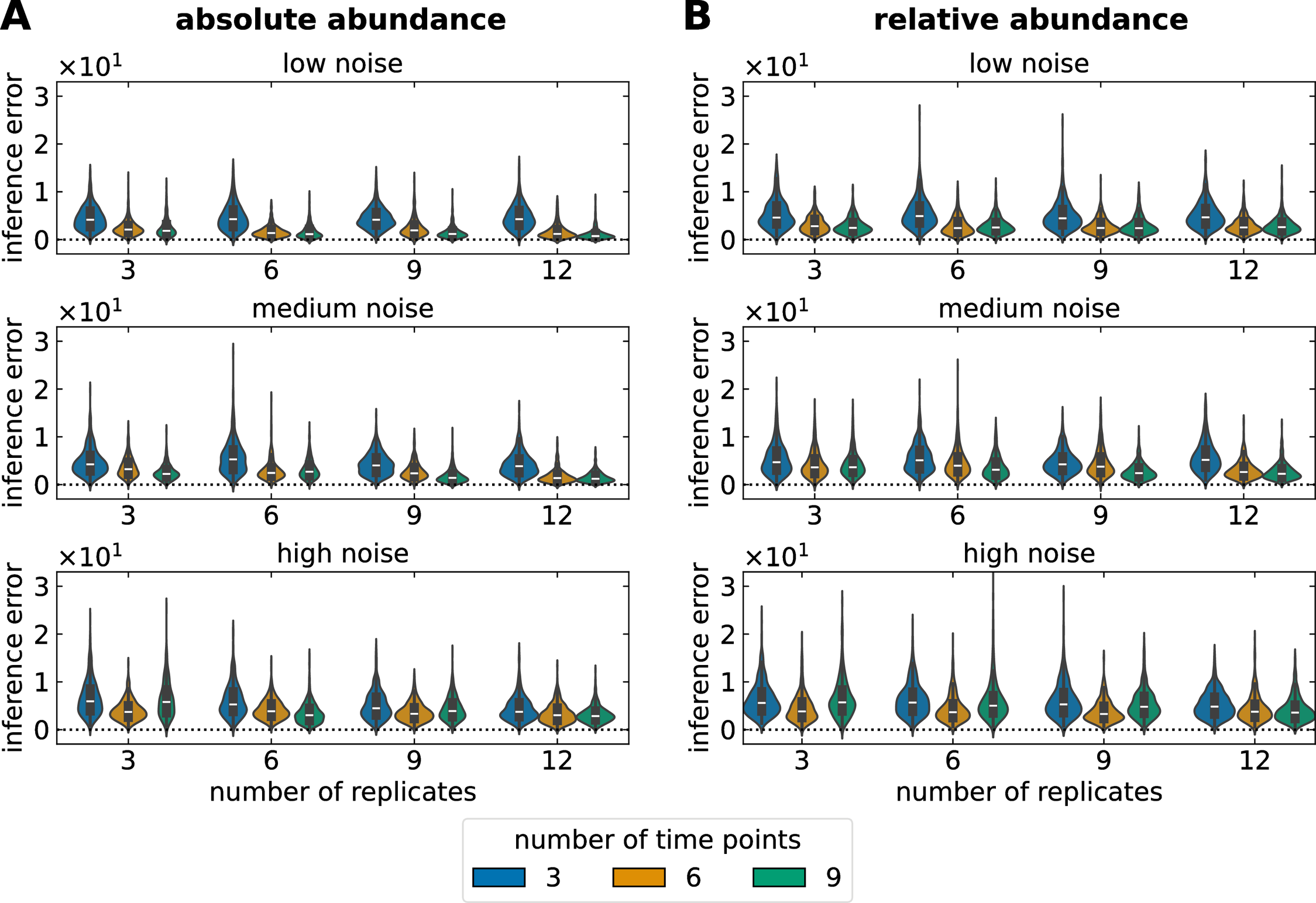

Supplement: S2 Fig — We inferred all parameters from simulated data as shown in Fig 2. To show the effect of noise on all parameters, we computed the L-2 norm of relative errors of the parameters (Table 2). We simplified the nuances of empirical noise assuming a scenario where all microbial abundances are affected proportionally. Concretely, a uniform noise distribution was shared among all microbial types and constant through time. For low noise, data could be altered by up to ±5%, while for medium and high noise, by up to ±10% and ±20%. Noise was sampled independently for each microbial type at each time point, affecting their absolute abundance from which relative abundances were computed. The data underlying this figure can be found in https://doi.org/10.5281/zenodo.13958305. (TIF) [file pbio.3002913.s002.tif]
