# Supplementary material for: Stochastic models allow improved inference of microbiome interactions from time series data
Source: PLoS Biol. 2024 Nov 21;22(11):e3002913. doi: 10.1371/journal.pbio.3002913 (PMC11620570; doi:10.1371/journal.pbio.3002913)
Supplement: Attachment — Submitted filename: review-PBIOLOGY-D-24-00520.pdf [file pbio.3002913.s003.pdf]

Report on manuscript PBIOLOGY-D-24-00520_R1

The manuscript "Inferring interactions from microbiome data" by Zapièn-Campos, Bansept, and Traulsen presents general methodology for parameter inference of stochastic mechanistic models from microbial abudance data using moment approximations. While the paper focusses on inference of a three-species Lotka-Volterra model, the presentation of the method is general enough that the study is relevant to a wide-range of applications. The paper is likely to be a valued contribution and is in my view worthy of publication, however I have several comments (in no particular order) that I would like to see addressed.

1. Given the intended audience of the paper, the decision to focus on two low-state-count systems is appropriate. However, the paper requires further discussion regarding the applicability of the method. For example: how many states is too many? How many time points? What if data are noisy?
2. The decision to neglect extrinsic noise is, in my opinion, an oversight that requires justification.
   (a) Inclusion of extrinsic/measurement noise would, I expect, render many systems practically non-identifiable (even though, as the authors verify, the system may be structurally identifiable).
   (b) Further on this comment, I would like to see further details on the nature of the data considered. Is it expected to be very noisy? Is it continuous or discrete (i.e., seq-count data)?
3. The authors state (Line 207) that their approach deals "by design" with "longitudinal (time series) data" and that it is possible to analyse snalshot data. However, as far as I can see the moment approach implicitly assumes that the data are snapshot: nowhere do the authors consider correlations between time-points.
4. Can the authors provide some further guidance as to what moment closure should be applied, and how it could be expected to affect results? Furthermore, it could be worth at least mentioning software tools that automatically construct closed systems of moment equations that can then be solved numerically from a given reaction network (i.e., `MomentClosure.jl`).
5. Can the authors provide justification for using ABC with the absolute-value based discrepancy metric?
   (a) How do the results compare to, i.e., a surrogate-based inference method? Since the authors obtain a set of first- and second-order moments, they could approximate the likelihood using a parametric distribution and apply any likelihood-based inference technique. At least to "tune" or initialise an ABC approach at a later stage, I believe this would significantly improve the computational feasability of the approach; particularly as the authors are aiming to present the approach in *PLOS Biology*, to a biological audience.
   (b) The choice of an absolute-value discrepancy is unusual. Furthermore, for many models I would not expect that the second-order moments be weighted equivalently to the first-order moments. This is for two reasons: first, there are n first order moments, say, and n^2 second order moments. Secondly, the second order moments are on a completely different scale, with units of abudance squared. Have the authors compared results to a discrepancy based on the *relative* difference between observed and modelled moments?
6. (Minor) If the authors work with a continuum model (i.e., an SDE), can they not just rescale or transform the system to be in terms of relative abundance to avoid inferring the initial conditions?
7. (Minor) I believe it disingeneous to refer to least-squares type inference (refs 5 and 8) as "best guess". Do the authors mean to refer to inference using only the mean-field model? Would these results be equivalent to the present approach if only the closed system of first-order moments is considered?
8. (Minor) Could the authors provide more detail between lines 67 to 73 as to why we need the number of parameters to be equal to the number of equations/states? Should this discussion not also include reference to the number of observation points (time points) if the goal is to ensure that the parameter to measurement relation can be bijective?
9. (Minor) The authors should mention other approximations, such as the linear noise approximation, that are widely used in this context and may be equivalent in certain regimes.
