# Supplementary material for: Stochastic models allow improved inference of microbiome interactions from time series data
Source: PLoS Biol. 2024 Nov 21;22(11):e3002913. doi: 10.1371/journal.pbio.3002913 (PMC11620570; doi:10.1371/journal.pbio.3002913)
Supplement: Attachment — Submitted filename: response_to_reviewers.pdf [file pbio.3002913.s004.pdf]

We thank the reviewers for their detailed and constructive feedback. We believe its implementation now leads to a clearer delivery of the message, improved readability, and broader discussion. Please find below our point-by-point reply. We leave comments in black and our replies in blue.

Reviewer 1.

The manuscript "Inferring interactions from microbiome data" by Zapién-Campos, Bansept, and Traulsen presents general methodology for parameter inference of stochastic mechanistic models from microbial abundance data using moment approximations. While the paper focuses on inference of a three-species Lotka-Volterra model, the presentation of the method is general enough that the study is relevant to a wide-range of applications. The paper is likely to be a valued contribution and is in my view worthy of publication, however I have several comments (in no particular order) that I would like to see addressed.

1. Given the intended audience of the paper, the decision to focus on two low-state-count systems is appropriate. However, the paper requires further discussion regarding the applicability of the method. For example: how many states is too many? How many time points? What if data are noisy?

Thank you for the comment. In the updated and substantially extended manuscript, we include new figures to illustrate the effect of the number of replicates, number of time points, and noise on our inference workflow. We use the Lotka-Volterra model with 3 microbial types as a case study. Concretely, the new **Fig 4** shows the effect on a single parameter, and the new **Fig Sup 2** shows the effect on all inferred parameters. Accompanying these figures, we extend the Results, Discussion, and IPython Notebooks.

We assumed that absolute abundances are affected by multiplicative noise sampled (i.e. in terms of a percentage), at each time point for each microbial type, from a uniform distribution in the domain (0.95, 1.05) for low noise, (0.9, 1.1) for medium noise, and (0.8, 1.2) for high noise. Our results show how the parameter uncertainty caused by noise can be reduced with a larger number of replicates or time points. Such improvement eventually saturates. Thus, **Fig 4** illustrates how determining the number of time points and replicates will depend on the amount of noise and dynamics captured.

Alongside comment 11 of reviewer 2, we highlight that our method is not limited by the number of states. In fact, this is one of the benefits of tracking statistical moments: a stochastic description of a large system without compromising on computational burden.

2. The decision to neglect extrinsic noise is, in my opinion, an oversight that requires justification.

2.1 Inclusion of extrinsic / measurement noise would, I expect, render many systems practically non-identifiable (even though, as the authors verify, the system may be structurally identifiable).

Thank you for raising this important point. We address the effect of noise in our reply to comment 1.

2.2 Further on this comment, I would like to see further details on the nature of the data considered. Is it expected to be very noisy? Is it continuous or discrete (i.e., seq-count data)?

We do not assume specific properties of data. Instead, our workflow only requires a table of absolute or relative abundances. The pre-processing leading up to that table could be considered in the workflow if it introduces artifacts that need to be considered as measurement noise. We mention this in the updated manuscript (lines 142-144) and highlight recent work comparing the artifacts introduced by common microbiome sequencing methods (Rausch et al, 2019).

3. The authors state (Line 207) that their approach deals "by design" with "longitudinal (time series) data" and that it is possible to analyze snapshot data. However, as far as I can see the moment approach implicitly assumes that the data are snapshot: nowhere do the authors consider correlations between time-points.

Thank you for raising this point. We aimed to say that data is a sequence of observations (snapshots) with time order, while the model that we derive is continuous. We clarify this point in the text now (**Fig 1**).

4. Can the authors provide some further guidance as to what moment closure should be applied, and how it could be expected to affect results? Furthermore, it could be worth at least mentioning software tools that automatically construct closed systems of moment equations that can then be solved numerically from a given reaction network (i.e., MomentClosure.jl).

Thank you for pointing this out. We answer this question as part of question 6 of reviewer 2. In addition, we thank the reviewer for pointing us to the MomentClosure.jl software, which we mention in the updated manuscript.

5. Can the authors provide justification for using ABC with the absolute-value based discrepancy metric?

5.1 How do the results compare to, i.e., a surrogate-based inference method? Since the authors obtain a set of first- and second-order moments, they could approximate the likelihood using a parametric distribution and apply any likelihood-based inference technique. At least to “tune” or initialize an ABC approach at a later stage, I believe this would significantly improve the computational feasibility of the approach; particularly as the authors are aiming to present the approach in PLOS Biology, to a biological audience.

Thank you for pointing into that direction. Using likelihood-based methods is indeed a promising avenue. If a stochastic model has analytic solution this corresponds to its likelihood. However, because most models do not have analytic solutions, we considered that likelihood-free (ABC) methods are useful for our proof-of-concept work. Alternatively, likelihood-based methods require an assumed pseudo-likelihood. We consider this a promising avenue, but also requiring further investigation beyond our proof-of-concept – to optimize the computational feasibility as the comment rightly suggests. We added these points in the updated manuscript (Methods and Discussion).

5.2 The choice of an absolute-value discrepancy is unusual. Furthermore, for many models I would not expect that the second-order moments be weighted equivalently to the first-order moments. This is for two reasons: first, there are n first order moments, say, and n2 second order moments. Secondly, the second order moments are on a completely different scale, with units of abundance squared. Have the authors compared results to a discrepancy based on the relative difference between observed and modelled moments?

Thank you for raising this point. We apologize for the lack of clarity about this. In the updated manuscript we include a new **Fig Sup 1** comparing the effect on the inference of using non-rescaled moments vs. rescaled moments.The rescaling we chose was to take the square root of larger moments. For absolute abundance, these were 2nd-order moments and co-moments, while for relative abundance data the 1st-order instead. **Fig Sup 1** shows that using non-rescaled moments achieves better results, i.e. less uncertain parameter values. The reason might be that among the larger moments some are enough to sufficiently constrain the inference, indirectly reducing the error of smaller moments. This is encouraging, because if rescaling was needed more degrees of freedom would have to be considered.

6. (Minor) If the authors work with a continuum model (i.e., an SDE), can they not just rescale or transform the system to be in terms of relative abundance to avoid inferring the initial conditions?

Thank you for the suggestion. We considered the possibility of directly deriving relative abundance equations at an early stage, however, we decided to maintain the scaling factor as well. The reason is that this rescaling factor is an important parameter to obtain in certain cases, e.g. in Wang, Y, et al, 2024. We mention this in the updated manuscript. In addition, our derivation tried to be as textbook-similar as possible, until the point where transformations to relative abundance and a scaling factor were derived.
Regarding how relative abundance equations could help to avoid inferring initial conditions, we will thank further elaboration from the reviewer. We might be misunderstanding this, but our interpretation is that any system of equations requires an initial condition regardless of it being expressed in absolute or relative abundance terms.

7. (Minor) I believe it disingenuous to refer to least-squares type inference (refs 5 and 8) as “best guess”. Do the authors mean to refer to inference using only the mean-field model? Would these results be equivalent to the present approach if only the closed system of first-order moments is considered?

We apologize for the confusing terminology. With “best guess” we referred to the inference approach employed in ref 8, which is linear regression (single point estimation) as opposed to Bayesian inference (distribution estimation). ”We changed “best-guess” to “linear regression” in the updated manuscript. Thank you for this suggestion!

The use of mean-field models in refs 5 and 8 is another topic altogether, being equivalent to our workflow with only the first-order moments and higher moments being approximated as the products of first order moments.

8. (Minor) Could the authors provide more detail between lines 67 to 73 as to why we need the number of parameters to be equal to the number of equations/states? Should this discussion not also include reference to the number of observation points (time points) if the goal is to ensure that the parameter to measurement relation can be bijective?

Thank you for the comment. As rightly pointed out by the reviewer, the identifiability of parameters not only depends on the relation between the number of parameters and the number of equations but also on the number of time points. We have rewritten those lines and expanded the explanation in the section “The advantages of our workflow for inference”.

9. (Minor) The authors should mention other approximations, such as the linear noise approximation, that are widely used in this context and may be equivalent in certain regimes.

Thank you for the suggestion. We have answered this in comment 7 of reviewer 2.

Reviewer 2.

The manuscript is very well written. The problem tackled is important and the approach is to my knowledge new for microbial time series.

Thank you for these positive comments.

The authors propose to use the equations for the first moments of the distribution of microbial abundances in replicate communities, those equations are derived from a master equation of the logistic model or the generalized Lotka-Volterra model. Parameter fitting is performed with a method called Approximate Bayesian Computation - Sequential Monte Carlo.

Major comments:

1. Only three different in silico systems have been tested. I would have appreciated a more extensive study to determine under which conditions model parameters can be inferred by the method. One would eventually want to be able to give instructions to experimentalists to tell for instance how densely sampled the system should be and how many replicates we need to be able to rely on this method.

Thank you for raising these important points. We address the effect of the number of replicates and time points in our reply to comment 1 of reviewer 1. In addition, in the updated manuscript we justify the focus on logistic growth and Lotka-Volterra as case studies to exemplify our inference workflow; in particular, that these two models often appear in studies of microbial communities.

2. Additionally, it is important to test the method on time series which are at steady state. It is often the case that for in vivo time series, we only have access to fluctuations around the steady state.

Thank you for raising this concern. We are aware that microbiome time series often show quasi-steady dynamics. However, making general statements about the performance of parameter inference methods on quasi-steady data is not straightforward, as inference exploits “active” dynamics to estimate the parameters (Cao, H T, et al, 2017). We highlight this fact in the updated manuscript and argue that microbiomes can be “activated” e.g. by perturbing its composition (page 12).

3. Comparison with other method could also be done. Could we rely on the method of Stein et al, 2013 with one time series (instead of averaging) and get the same parameters? It is said that one advantage of the proposed workflow is that "the dynamics of the moments uses more information contained in the data", but it is not needed to average the data to apply the method of Stein et al. for instance (or do I miss something?). If replicates are available, we could apply Stein's method multiple times and then compute the averages of the parameters and their variabilities. If we do so, would we get the same result as with your method?

Thank you for the suggestion (related to comment 1 of reviewer 3). In the updated manuscript we include a new **Fig 3**, which shows the comparison between our workflow and Stein’s method on simulated data. Our results indicate that our workflow is able to “locate” the true value of the Lotka-Volterra parameters even when Stein’s method does not. The improvement seems to be given by the extra statistical information that our statistical moments track.

Applying Stein’s method to individual replicates and averaging the resulting parameters could lead to artifacts. Concretely, as demographic noise could lead to large abundance differences between replicates, Stein’s method could overfit them. Thus, when replicates are available, taking their mean to average-out those differences would be preferred. This was our approach in **Fig 3**. Our workflow computes far more statistical moments than Stein’s method, some of which might capture features that improve the identifiability/inference of the parameters.

4. It seems that the method can only be applied if the initial conditions are identical in the replicates. This seems hard to realize for in vivo microbial communities. Are they ways to circumvent this limitation?

As rightly pointed out by the comment, our workflow requires replicates. More concretely, replicates are single realizations of the same stochastic process with equal environmental and initial conditions. In that line, we chose the OMM[12] dataset as a well-controlled experiment, where such conditions were enforced to the best possible extent. We emphasize this point in the updated manuscript (page 12) and acknowledge that obtaining true replicates can be an issue for out-of-lab samples.

Follow-up research is needed to determine if perturbation experiments, where the microbiome composition is changed from a stationary state, can be used to generate replicates, for example, after antibiotics usage or microbiome transplantation. Alternatively, from a theoretical perspective, one could model the microbial types at higher taxonomic levels, where microbial compositions are more similar to each other among hosts **(**Rausch et al., 2019**)**.

5. [this question is similar to the previous one] It is not clear to me which type of experimental data can be

aggregated and modeled as P(n,t). Can they be coming from different environmental conditions, different initial conditions, different species compositions? Or do we need very well controlled experimental conditions?

Answered in question 4 above.

6. Line 284: I would want to know if in typically experimental microbial time series the approximation \sim is true. Is this easy to check?

Thank you for raising this important point. Several approximations of third-order moments have been used in the literature (Kuehn, C, 2016), some of which have a more grounded basis than others. In our work, we propose an approximation where covariance is the key concept. First, a pair of microbial types is formed and a third one is left alone; our approximation requires the covariance of the pair and the single one being approximately zero. Note that three possible ways exist to accommodate the three microbial types, where some might indeed have a covariance close to zero. Despite the fact that our approximation is for illustration only, its validity can be checked in each individual dataset, considering alternative approximations as well **(**Kuehn, C, 2016**)**.

Technically, validating our approximation involves computing the covariance of each triplet from the time-series data and confirming that the number is close to zero. A priori, from the definition of covariance we see that this number is close to zero if either or both of the following are true:
i) the difference between the mean abundance of the single type and its individual replicates is close to zero,
ii) the difference between the mean abundance of the pair of types and its individual replicates is close to zero.
This can be true in cases where the microbiome dynamics among replicates are similar to each other (Eberl, C., et al, 2020 suggests this could indeed happen). We include part of this explanation in the updated manuscript (page 19).

7. For the mice experimental data, why didn't you try fitting a generalized Lotka-Volterra model? From the conclusions of Grilli, Nature Communications 2020 and Descheemaeker eLife 2020, this makes sense as the time series analyzed in those papers concluded that the characteristics of the fluctuations around steady states can be explained with a stochastic logistic model with linear noise, and therefore no pairwise interactions (however transient dynamics is very limited in the time series analyzed).

Thank you for pointing this out. Firstly, a logistic growth model was just used to illustrate the workflow, as it captures the most striking features of the OMM^{12} dataset, transient growth and stationary stages. Secondly, there is a large difference in the number of parameters involved, from 3*12 + 1 = 37 for logistic growth to 12*12 + 12 = 156 for Lotka-Volterra. As a consequence, the computational time needed to infer Lotka-Volterra parameters is much longer. The Bayesian inference step must be optimized further, as despite ABC not needing a likelihood function, the computational time is large. We are investigating other methods currently.

We updated our manuscript to better state the reasons behind our modelling choices and included references to the linear noise approximations of Grilli, J, 2020 and Descheemaeker, L, et al, 2020 as alternative models to consider and that can be accommodated in our parameter inference framework (page 13).

Minor comments/questions:

8. Line 141: I do not know what identifiability tableaus means.

We apologize for the lack of clarity. GenSSI's approach transforms the system of ODEs to series around each time point (Chis, OT, et al, 2011; Walter, E, et al, 1982). The coefficients of such series include the parameters and have the property of containing all the information required to go from input to output. An identifiability tableau is used to summarize how many parameters are identifiable. In particular, columns correspond to the number of parameters and rows to the number of non-zero coefficients of the series (Chis, OT, et al, 2011). We have included a synthesized explanation of this in the manuscript (pages 8-9).

9. For equation (10a), I guess N means the maximal number of microbes in the system. If that is correct, I would say it explicitly.

Thank you for the suggestion, we have now written that N is the maximum number of microbes in a host.

10. In equation (10b) it is not directly obvious to me why the death rate would decrease in a larger population (i.e. a population with a larger N).

Thank you for raising this point. We rewrote the paragraph immediately below to clarify it. Note that overall, the death rate is proportional to the fraction of microbes of type *i* (n_i / N), not just *N*, and we assume φ_i is small. Thus, far from carrying capacity (*N*), the death of microbes is rare. This is in fact intended, as in a typical logistic model, no death rate is assumed, and equilibrium is reached solely at carrying capacity where growth is zero. Building on this concept, our stochastic model assumes that the death rate only plays a role once the added population size (Σn_j) approaches the shared carrying capacity. Once $\Sigma n_j = N$, all growth rates are zero, equation (10a), and all death rates are greater than zero, equation (10b). Thus, only death can happen. Once $\Sigma n_j < N$, death rates have a similar or smaller magnitude than growth rates. Thus, mostly growth will happen. This "death-birth process" close

to carrying capacity allows populations to slowly converge towards equilibria determined not only by birth (heavily influenced by the initial condition), but also by death and immigration rates.

11. For the simulated time series, you used N=10^5. Can you simulate realistic numbers? For human gut microbial communities, the number of microbes is much larger.

Thank you for this observation. We used N=10^5 as a minimum realistic microbiome (*C. elegans*, Vega, NM, et al, 2017). In the updated manuscript we emphasize that larger numbers can be used (Tables 1 and 2), especially since the equations for the statistical moments can deal with large numbers without an explosion of the computational burden - as we do not need to simulate individual microbes.

Reviewer 3.

The manuscript entitled "Inferring interactions from microbiome data", provides a significant contribution to the field of theoretical microbiology. The authors highlight the limitations of fitting deterministic models to averaged data, which often result in the loss of valuable information. To address these limitations, the manuscript proposes a stochastic model to infer distributions of interaction parameters that best describe biological experimental data.

Starting with a stochastic model, the authors derive dynamical equations for not only the average but also higher statistical moments of microbial abundances. These equations are utilized to infer distributions of interaction parameters, thereby improving identifiability and precision in describing the experimental data. Compared to existing approaches, this method offers a more comprehensive understanding of interactions in microbiomes.

Overall, I have really enjoyed reading this manuscript. The work presents a compelling and innovative approach to study microbial interactions within microbiomes. The problem of integrating theoretical models with experimental data in microbiome research is well-motivated, and the proposed method represents a significant advancement in addressing this challenge. I am in principle in favor of publication, but subject to the following major and minor suggestions for improvement.

Thank you for these very positive comments.

Major suggestions

1. While the manuscript significantly demonstrates the effectiveness of combining Bayesian inference and stochastic modeling to infer interactions from microbiome data, it could benefit from a clearer explanation of how this approach surpasses classical deterministic methods. Additionally, it would also be more informative to compare and contrast the advantages and disadvantages of the proposed model with existing models in the field.

Thank you for the suggestion (related to comment 3 of reviewer 2). To address this, in the updated manuscript we compare the linear regression approach of Stein et al, 2013, against our workflow in the new **Fig 3**. We show that our workflow consistently “locates” the true parameter values, even in cases where Stein’s method does not.
In addition, we discuss alternative models to our logistic and Lotka-Volterra models and their model comparison.

2. The impact of underlying network structures on microbial community dynamics is well-recognized. It would be beneficial to explore the applicability of this method to a range of network structures, including temporal networks (Li et al., Science 358: 1042-1046, 2017; Nat. Comm. 11: 2259, 2020) and multi-layer networks (Wang et al., J. R. Soc. Interface 20: 20220752, 2023). Such an analysis could provide a more comprehensive understanding of the method's potential and limitations in various network contexts within microbial communities.

Thank you for this suggestion. In our updated manuscript (page 13) we mention how time-dependent interactions and interaction assortment could be accommodated in our workflow; in particular, through the transition rates. Given that the dynamical moments derive from the master equation, considering time-dependent interactions does not affect our mathematical derivation. Meanwhile, interaction assortment, such as in multi-layer networks, can be included as in our Lotka-Volterra model, where positive and negative interactions are separated. A similar separation can be made based on the microbial identity.

3. In addition to pairwise interactions, it is important to consider other forms of interactions in this stochastic model, such as higher-order interactions and time delay interactions (Yang et al. Nat. Ecol. Evol. 7: 1610-1619, 2023). Discussing these interaction types would enrich the model and provide a more comprehensive understanding of microbial community dynamics. It is also meaningful to discuss the effects of this stochastic model on different ecological traits, such as stability and reactivity. Exploring these implications would provide valuable insights into the broader ecological implications of this stochastic model.

Thank you for this suggestion. Alongside our previous answer, we mention higher-order interactions and time-delayed interactions in our updated manuscript. Both cases are similarly incorporated through the transition rates. Other ecological traits, beyond the composition, require research beyond our proof-of-concept inference workflow.

Minor suggestions

4. The title appears somewhat confusing. A more detailed and descriptive title would enhance the clarity of this manuscript.

Thank you for pointing this out. We have changed the title “Inferring interactions from microbiome data” to the more descriptive “Inferring microbiome interactions from distributions of time series replicates”.

5. Discussions on the limitations of the stochastic model are not sufficiently thorough and could be expanded to provide a more comprehensive assessment.

We apologize for the limited self-criticism of our models. We have extended the discussion on the limitations of the included models and (related to comment 7 of reviewer 2) point to alternative models that might explain the same experimental observations.

6. The figure captions should include specific parameter values to enhance the clarity and reproducibility of the results.

Thank you for the suggestion, we agree that including the parameter values is paramount. We have rewritten the captions of **Fig 2** and **Fig 5** to indicate that their numerous parameters are listed in Tables 1-4 and 6 (for **Fig 2**) and Table 5-6 (for **Fig 5**).

7. A more detailed discussion is needed regarding the application of this model, including its practical implications in real-world scenarios.

Thank you for the suggestion. Gathering the feedback of the reviewers, we now explore practical aspects that include the effect of the number of samples, number of replicates, and amount of measurement noise (new **Fig 4**). A detailed description of these aspects is provided in comment 1 of reviewer 1.

Apart from this, I am happy to congratulate the authors for their inspiring work and reiterate my recommendation for acceptance in PLoS Biology.

References

Cao, H. T., Gibson, T. E., Bashan, A., & Liu, Y. Y. (2017). Inferring human microbial dynamics from temporal metagenomics data: Pitfalls and lessons. *BioEssays*, *39*(2), 1600188.

Chis, O. T., Banga, J. R., & Balsa-Canto, E. (2011). Structural identifiability of systems biology models: a critical comparison of methods. *PloS one*, *6*(11), e27755.

Descheemaeker, L., & De Buyl, S. (2020). Stochastic logistic models reproduce experimental time series of microbial communities. *Elife*, *9*, e55650.

Eberl, C., Ring, D., Münch, P. C., Beutler, M., Basic, M., Slack, E. C., ... & Stecher, B. (2020). Reproducible colonization of germ-free mice with the oligo-mouse-microbiota in different animal facilities. *Frontiers in microbiology*, *10*, 2999.

Grilli, J. (2020). Macroecological laws describe variation and diversity in microbial communities. *Nature communications*, *11*(1), 4743.

Kuehn, C. (2016). Moment closure—a brief review. *Control of self-organizing nonlinear systems*, 253-271.

Rausch, P., Rühlemann, M., Hermes, B. M., Doms, S., Dagan, T., Dierking, K., ... & Baines, J. F. (2019). Comparative analysis of amplicon and metagenomic sequencing methods reveals key features in the evolution of animal metaorganisms. *Microbiome*, *7*, 1-19.

Vega, N. M., & Gore, J. (2017). Stochastic assembly produces heterogeneous communities in the Caenorhabditis elegans intestine. *PLoS Biology*, *15*(3), e2000633.

Walter, E., & Lecourtier, Y. (1982). Global approaches to identifiability testing for linear and nonlinear state space models. *Mathematics and Computers in Simulation*, *24*(6), 472-482.
Wang, Y., Chen, S., Hu, J., & Zhou, D. (2024). Determining cell population size from cell fraction in cell plasticity models. *arXiv preprint arXiv:2405.04557*.
