# Supplementary material for: Stochastic models allow improved inference of microbiome interactions from time series data
Source: PLoS Biol. 2024 Nov 21;22(11):e3002913. doi: 10.1371/journal.pbio.3002913 (PMC11620570; doi:10.1371/journal.pbio.3002913)
Supplement: Attachment — Submitted filename: reply_to_reviewers.pdf [file pbio.3002913.s005.pdf]

We thank the reviewers for the comments on the updated manuscript. After addressing them, we believe the new version is more precise. Please find below our point-by-point reply. We leave comments in black and our replies in blue.

Reviewer 1.

The authors have largely addressed my original comments, however I have two minor points that remain.

1.1 The authors claim that the method is not limited by the number of states. I can't see how this is true. If the authors have, say, 20 states, with covariances and means (first and second order moments), the authors would have to analyse a system with, effectively, 200+ states. Can the method really handle this?

Thank you for this comment. Please excuse us if we interpret the comment differently, but by 'states' we understand that the reviewer refers to what we call 'microbial types'. With 20 microbial types there would be 20 equations for first moments and 200 equations for 2nd order moments. This is indeed a large number of differential equations. However, one of the advantages of our approach based on statistical moments is that 1) many of the equations are linear (except those where moment closure is applied), and 2) that conventional ODE solvers can be used. As proof-of-principle, we show the parameter inference of a dataset with 12 microbial types (Figure 5) where a system of 12+144 equations is solved at each iteration of the Approximate Bayesian Computation. Beyond this proof-of-principle, further optimizations would reduce the computational demands for cases with more microbial types (lines 79-81).

2.1 The authors claim that they cannot use a likelihood based method unless the SDE admits an analytical solution. However, I would expect the moment-matching ABC approach to perform similarly (but much more slowly) to an approximate likelihood-based approach where a Gaussian likelihood with a matched mean and covariance is used.

Thank you for raising this point. We agree that using a pseudo-likelihood approach would perform similarly in many cases while reducing computational time. For example, in Figure 5 the posterior distribution of the parameters obtained by ABC is normal. In the updated manuscript we emphasize that using a pseudo-likelihood approach is a promising direction to reduce the computational time while benefiting from well-establish Bayesian methods (lines 91-92).

Reviewer 2.

Overall, I believe the manuscript has improved, and most of the comments have been addressed. However, I still think there are some areas that require further refinement.

We thank the reviewer for their positive view of the updated manuscript.

2.1 Regarding the recommendations for experimentalists, would you suggest that 4 replicates and 3 time points are sufficient? I would assume it is also important to specify the intervals between time points, as well as emphasize that the time points should be captured during the transient dynamics phase. From Fig. 4, you conclude that it is more beneficial to increase the number of time points than to increase the number of replicates. However, in the extreme case where additional time points are only taken at steady-state, I suspect it might be more advantageous to increase the number of replicates. I believe the conclusion drawn from Fig. 4 could be more nuanced.

Thank you for this important comment. Figure 4 suggests that increasing the number of time points might be more beneficial that increasing the replicates (more than 3 time points might be needed). However, each experimental dataset might present their own challenges. We agree that for a near to steady-state scenario, increasing the replicates would be more advantageous. We have expanded our discussion (lines 240-246) to include these nuances.

2.2 You mentioned that the method could be applied to snapshots. Could you provide an example where a single time point is sufficient to recover the parameters? Or did you mean that while the method could be applied to snapshots, it would not be effective?

Thank you for raising this point. We apologize for the lack of clarity. We mean that while the method could be used for snapshots it would not be as effective as using longitudinal data. We make this more clear in the updated manuscript (lines 259-260).

2.3 Is there any way to estimate the noise level in the OMM dataset, for instance? If so, this would strengthen Fig. 4, as you could argue that the noise level in the experimental dataset is comparable to the one for this dataset.

Thank you for raising this point. For the simulated dataset of Figure 4 we had to propose a model for the noise. However, in Figure 5 information about the functional form of the noise of the OMM[12] dataset is unknown. Thus,

we refrained from assuming a functional form of noise in Figure 5. Instead, we clarify that any knowledge of the noise can be incorporated in the model (lines 239-242).

2.4 Regarding Fig. 5C, I am uncertain whether measuring uncertainty as s.d.post/s.d.prior is the best approach for assessing the quality of the inference. I would prefer reporting the posterior standard deviation alone. If the prior has a large width, the ratio might decrease, but the actual uncertainty in the estimation would not. Or am I misunderstanding something?

Thank you for this observation. We use *s.d.post/s.d.prior* to rank the parameters according to how much certainty was gained through the inference. We agree that *s.d.post* would rank the most certain parameters in absolute terms, however, to compare parameters of different magnitude (e.g. growth vs. death rates), *s.d.post/s.d.prior* allow us to present the decrease of uncertainty as a relative quantity. Then, because all parameters of each type share the same prior (Table 5) we can compare their decrease of uncertainty too. We clarify these points in the updated manuscript (legend of Fig 5B).

Reviewer 3.

The authors have made great efforts in addressing my comments. I am happy to recommend the acceptance, but please check there are many question marks on the current version when preparing the final version.

We thank the reviewer for their positive comment. We apologize for the question marks in the former version which seem to have been caused by the compilation of the LaTeX file.
